# Posterior Reversible Encephalopathy Syndrome Secondary to CSF Leak and Intracranial Hypotension: A Case Report and Literature Review

**DOI:** 10.1155/2015/538523

**Published:** 2015-05-27

**Authors:** Tariq Hammad, Alison DeDent, Rami Algahtani, Yaseen Alastal, Lawrence Elmer, Azedine Medhkour, Fadi Safi, Ragheb Assaly

**Affiliations:** ^1^Department of Internal Medicine, University of Toledo Medical Center, 3000 Arlington Avenue, Toledo, OH 43615, USA; ^2^Department of Neurology, University of Toledo Medical Center, 3000 Arlington Avenue, Toledo, OH 43615, USA; ^3^Department of Neurosurgery, University of Toledo Medical Center, 3000 Arlington Avenue, Toledo, OH 43615, USA; ^4^Division of Pulmonary and Critical Care, Department of Internal Medicine, University of Toledo Medical Center, 3000 Arlington Avenue, Toledo, OH 43615, USA

## Abstract

Posterior Reversible Encephalopathy Syndrome (PRES) is a clinical neuroradiological condition characterized by insidious onset of neurological symptoms associated with radiological findings indicating posterior leukoencephalopathy. PRES secondary to cerebrospinal fluid (CSF) leak leading to intracranial hypotension is not well recognized etiology of this condition. Herein, we report a case of PRES that occurred in the setting of CSF leak due to inadvertent dural puncture. Patient underwent suturing of the dural defect. Subsequently, his symptoms resolved and a repeated brain MRI showed resolution of brain lesions. The pathophysiology and mechanistic model for developing PRES in the setting of intracranial hypotension were discussed. We further highlighted the importance of tight blood pressure control in patients with CSF leak and suspected intracranial hypotension because they are more vulnerable to develop PRES with normal or slightly elevated bleed pressure values.

## 1. Background and Importance

Posterior Reversible Encephalopathy Syndrome (PRES) is a clinical neuroradiological condition characterized by insidious onset of headache, altered mental status, seizures, and cortical blindness associated with findings indicating posterior leukoencephalopathy on imaging studies [1]. Although the condition was described earlier in the literature, the term (PRES) was initially coined in 1996 by Hinchey and his colleagues [1–3]. It has been mainly described in association with hypertensive crisis—particularly in the setting of renal failure, eclampsia, sepsis, and the use of immunosuppressant drugs such as calcineurin inhibitors [1]. With the increased awareness of PRES and widespread use of the imaging studies, there has been a tremendous increase in the reported cases associated with other conditions. Herein, we report a case of PRES in a 72-year-old man that occurred in the setting of cerebrospinal fluid (CSF) leak. The pathophysiology and mechanistic model for developing PRES in the setting of intracranial hypotension will be discussed as well.

## 2. Clinical Presentation

**Figure 1:**
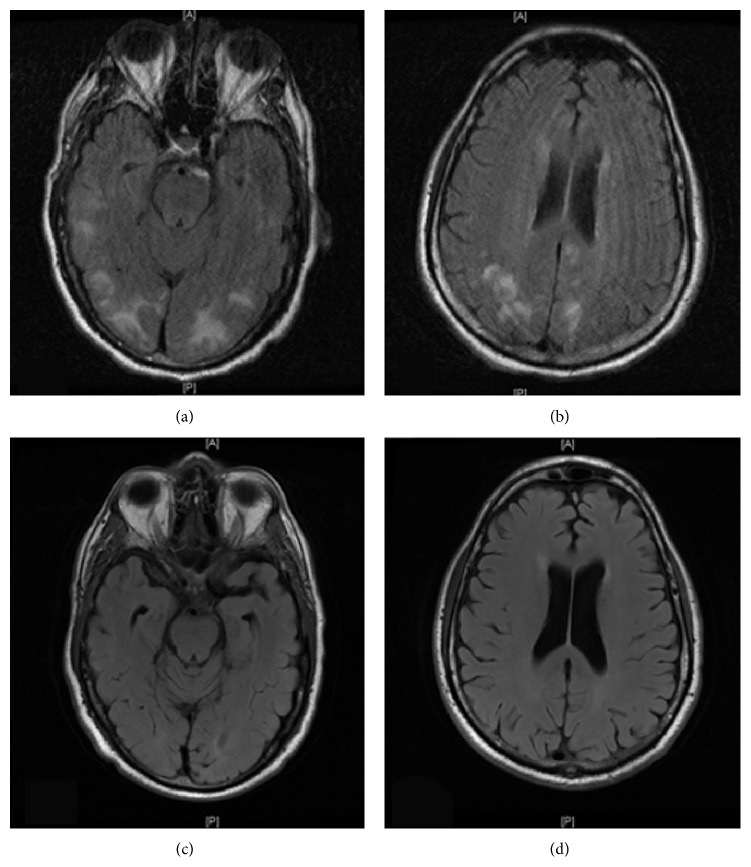
((a) and (b)) Brain MRI T2 FLAIR images show leptomeningeal enhancement and high signal intensity lesions in the subcortical white matter involving both hemispheres consistent with PRES. ((c) and (d)) Brain MRI T2 FLAIR images after 3 weeks show complete resolution of the lesions.

A 72-year-old man with history of controlled hypertension and chronic back pain underwent spinal fusion and laminectomy surgery from the third lumber to the first sacral vertebral bodies. Surgery was complicated by CSF leak due to inadvertent puncture of the dura that presented one week postoperatively with fluid collection at the site of the surgical incision. The patient was admitted to the hospital for further evaluation and management. He developed a moderate diffuse postural headache that was thought to be related to CSF leak. He was managed conservatively with analgesia and hydration and underwent a blood patch. An external lumbar drain (ELD) was placed to reduce the leak, speed up the healing at the dural puncture site, and monitor the quantity of the leak. A total of 1150 mL of fluid leak was recorded over the subsequent one week. On postoperative day fifteen, the patient developed severe occipital headache and transient vision loss for few minutes with deterioration of mental status, followed by tonic-clonic seizure. Blood pressure was elevated at the level of 170/100 mmHg. Seizure was controlled by lorazepam and the patient started on levetiracetam 500 mg bid. Brain computed tomography scan (CT) excluded intracranial bleeding. The patient did not regain consciousness completely after the seizure and continued to be confused for few hours. Hence, magnetic resonance imaging (MRI) was performed and showed leptomeningeal enhancement and high signal intensity lesions in the subcortical white matter involving both hemispheres consistent with PRES (Figures 1(a) and 1(b)). Magnetic resonance angiography and venography of the head and neck were negative. Electroencephalogram showed changes consistent with diffuse encephalopathy. CSF routine bacterial, fungal, and viral cultures were negative. Vasculitis workup was negative. Patient ultimately underwent suturing of the dural defect in view of the continuous CSF leak. His mental status and headache improved postoperatively. He did not develop further episodes of vision loss or seizure. Repeated brain MRI three weeks later showed resolution of the hyperintense lesions in the posterior white matter (Figures 1(c) and 1(d)).

## 3. Discussion

The pathophysiologic process underlying PRES is not well understood. In hypertensive emergency state, the extremely elevated mean arterial blood pressure (MAP) results in increased cerebral perfusion pressure (CPP) and brain hyperperfusion which subsequently leads to cerebral blood vessels autoregulatory failure and endothelial dysfunction [1, 4]. The hyperperfusion and endothelial dysfunction then cause increased capillary permeability and lead to brain tissue vasogenic edema resulting in the clinical and radiological manifestations of PRES [5]. On the other hand, preeclampsia and cytotoxic agents can cause direct endothelial toxicity and dysfunction leading to the manifestations above [1, 6].

**Figure 2:**
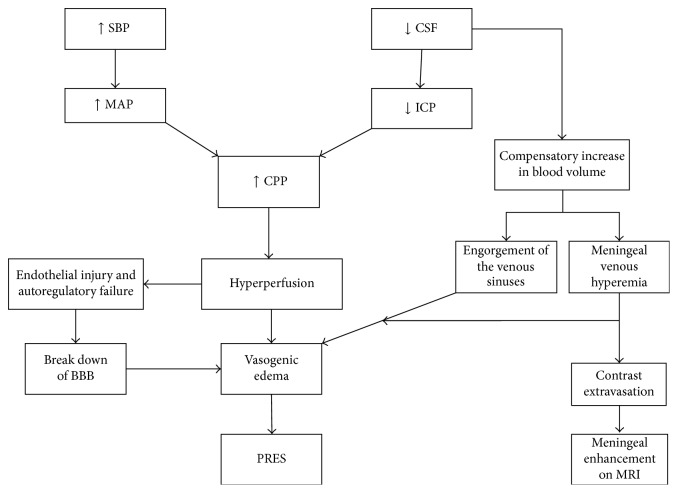
It represents a stepwise mechanistic explanation of pathophysiologic processes that result in vasogenic edema and PRES in context of CSF leak and intracranial hypotension. SBP: systolic blood pressure, CSF: cerebrospinal fluid, MAP: mean arterial pressure, ICP: intracranial pressure, CPP: cerebral perfusion pressure, BBB: blood brain barrier, PRES: Posterior Reversible Encephalopathy Syndrome, and MRI: magnetic resonance imaging.

In our patient, PRES was diagnosed based on typical clinical presentation and MRI finding after excluding central nervous system infections. The magnitude of blood pressure elevation was not convincing enough to consider it as the primary precipitant of PRES in this patient and there were no other known risk factors to be blamed. Therefore, we hypothesized that the moderate elevation in blood pressure associated with the intracranial hypotension secondary to the CSF leak caused a degree of hyperperfusion that precipitated PRES (see illustrative diagram, Figure 2).

Brain perfusion is dependent on systemic mean arterial pressure (MAP) and intracranial pressure (ICP). Either an increase in MAP or a decrease in ICP will result in an increase in cerebral perfusion pressure (CPP) and tacitly the brain perfusion. When the cerebral blood flow autoregulatory mechanisms are overwhelmed by the severe increase in the CPP, it results in hyperperfusion. This further leads to endothelial dysfunction at cellular level and subsequent vasogenic edema. Our patient has significant CSF leak of more than a liter over one week which resulted in decreased ICP and increased CPP.

Furthermore, as the volume inside the cranium is a fixed volume composed of CSF, blood, and brain tissue, any decrease in volume of one constituent must be compensated by an increase in volume of the other constituents [7]. In our patient, the CSF loss was not adequately compensated by increased production. Therefore, it was compensated by increase in brain tissue and blood volume. Increased intracranial blood volume resulted in venous stagnation and engorgement of venous sinuses. This further leads to increased venous hydrostatic pressure and vasogenic edema. The meningeal vessels engorgement explains the meningeal enhancement that was visualized in the MRI images of our patient during the disease (see illustrative diagram, Figure 2).

PRES associated with CSF leak has been reported previously in the literature particularly in the setting of epidural tap (see Table 1). In six cases, PRES developed post-CSF leak from epidural or spinal anesthesia in the setting of known situations to cause PRES like preeclampsia, cyclosporine, sepsis, and reversible cerebral vasospasm [8–13], whereas, in the other four cases, PRES developed post-CSF leak secondary to inadvertent puncture of dura during epidural anesthesia in the absence of precipitating factors [14–17]. Interestingly, Grelat et al. reported a case of recurrent PRES in a 69-year-old female patient following depletive CSF drainage for chronic adult hydrocephalus. Initially she developed PRES after therapeutic lumbar puncture. And four months later she developed similar episode after she underwent ventriculoperitoneal shunt for CSF drainage and lowering ICP which required “high pressure” shunt's valve reset to decrease the CSF drainage to treat the PRES episode [16].

## 4. Conclusion

In general, the prognosis of PRES is favorable if it is detected early and the precipitating factors are treated properly [1, 18]. In this case report and literature review, we aim to highlight three observations. First, we would like to highlight the possible association between PRES and intracranial hypotension secondary to CSF leak and overdrainage. Second, PRES should be early suspected in cases of CSF leak in the proper clinical context where the symptoms and signs such as resistant headache and visual changes could not be fully explained by intracranial hypotension alone as early diagnosis and treatment of the syndrome and underlying causes are crucial to prevent significant morbidity and mortality. Third, we suggest that blood pressure should be tightly controlled in patients with CSF leak and suspected intracranial hypotension because they are more vulnerable to develop PRES with normal or slightly elevated bleed pressure values. Whether this association represents a causal relationship is difficult to postulate at this juncture, and more case reports are required to validate these observations.

## Figures and Tables

**Table 1:** This table shows the reported cases in the literature of PRES in the setting of CSF leak.

Case	Age, sex	Cause of CSF leak	Associated risk factors	Highest BP	Treatment	Outcome
Torrillo et al. 2007 [8]	32, F	Epidural tap for labor	Preeclampsia	160/90	BP control	No residual deficit
Ho and Chan 2007 [9]	33, F	Spinal tap for CS	RCVS	140/80	MgSO4 for vasospasm	No residual deficit
Muñoz et al. 2009 [14]	36, F	Epidural tap	⋯	150/86	Blood patch	No residual deficit
Pradhan et al. 2009 [10]	34, F	Epidural tap for kidney transplantation	Cyclosporine	NA^*∗*^	Blood patch and surgical repair of dural tear	No residual deficit
Pugliese et al. 2010 [15]	41, F	Epidural tap for CS	⋯	NA^*∗*^	Blood patch	No residual deficit
Minai et al. 2011 [11]	Young, F	Epidural tap for CS	Sepsis	NA^*∗*^	Blood patch	No residual deficit
Orehek et al. 2012 [12]	26, F	Epidural tap for CS	Preeclampsia	180 s	Blood patch and BP control	Mild dysmetria
Grelat et al. 2014 [16]	69, F	Depletive LP and VPS	⋯	NA^*∗*^	Shunt control	Visual and motor deficit
Doherty et al. 2014 [17]	F	Epidural tap for CS	⋯	158/91	Blood patch	No residual deficit
Shah et al. 2014 [13]	62, F	Epidural tap for abdominal surgery	Ischemic colitis	190/80	Blood patch	Minor visual and memory deficit

CSF: cerebrospinal fluid. BP: blood pressure. F: female. CS: Caesarian section. RCV: reversible cerebral vasospasm. NA^*∗*^: not available, but the patient did not have severe hypertension.
